# "Booster" interventions to sustain increases in physical activity in middle-aged adults in deprived urban neighbourhoods: internal pilot and feasibility study

**DOI:** 10.1186/1471-2458-11-129

**Published:** 2011-02-23

**Authors:** Emma J Scott, Munyaradzi Dimairo, Daniel Hind, Elizabeth Goyder, Robert J Copeland, Jeff D Breckon, Helen Crank, Stephen J Walters, Amanda Loban, Cindy L Cooper

**Affiliations:** 1 Centre for Sport and Exercise Science, Faculty of Health and Well-being, Sheffield Hallam University, Collegiate Crescent Campus, Sheffield, S10 2BP, UK; 2 Clinical Trials Research Unit, University of Sheffield, Regent Court, 30 Regent Street, Sheffield, S1 4DA, UK; 3 School of Health and Related Research (ScHARR), University of Sheffield, Regent Court, 30 Regent Street, Sheffield, S1 4DA, UK

## Abstract

**Background:**

Systematic reviews have identified a range of brief interventions which increase physical activity in previously sedentary people. A randomised controlled trial is needed to assess whether providing motivational interviewing, three months after giving initial advice, sustains physical activity levels in those who recently became physically active. This paper reports the results of an internal pilot study designed to test the feasibility of the study in terms of recruitment, per protocol delivery of the intervention and retention at three months.

**Methods:**

Participants were: aged 40-64 years; resident in deprived areas of Sheffield, UK; and, had recently become physically active as a result of using a brief intervention following an invitation from a mass mailout. Interventions: Motivational Interviewing 'boosters' aimed at sustaining change in physical activity status delivered face-to-face or over the telephone compared with no further intervention. Outcomes of the feasibility study: recruitment of 60 participants from mailout of 3,300; retention of 45 participants with 3-month follow-up accelerometry measurements; 70% of those randomised to boosters receiving intervention per protocol. Sample size and power were recalculated using the accelerometry data collected.

**Results:**

Forty-seven participants were randomised (78% of the feasibility target); 37 participants were retained at three months, 29 with at least four days of accelerometry data (64% of the feasibility target); 79% of those allocated boosters received them per protocol (surpassing the feasibility target). The proposed sample size of 600 was confirmed as appropriate and power is expected to be sufficient to detect a difference between groups.

**Conclusions:**

The main study will continue with the original recruitment target of 600 participants but to ensure feasibility, it is necessary to increase recruitment and improve the numbers of those followed-up who have evaluable data. Strategies will include increasing the number of initial invitations sent out and improving the training of research assistants and participants in the positioning of the accelerometer.

**Trial Registration:**

ISRCTN: ISRCTN56495859, ClinicalTrials (NCT): NCT00836459

## Background

Participation in regular physical activity is associated with reductions in all-cause mortality [1] and decreased risk of cardiovascular disease [2] and some forms of cancer [3]. Additionally, there is a role for regular exercise in both the prevention and treatment of hypertension [4], type 2 diabetes [5] and obesity [6]. From a psychological perspective, regular physical activity has been shown to relieve anxiety [7], improve mood [8] and reduce depressive symptoms [9]. The majority of the British population, however, are not active enough to experience these benefits [10]. The primary aim of intervention programmes is to encourage participants to increase their activity sufficiently to meet the current recommended levels for health benefit [11].

A recent systematic review established that brief interventions in primary care can increase physical activity levels [12] and identified a sufficient evidence base for the UK National Institute of Health and Clinical Excellence (NICE) to recommend their use [13]. The review, however, also identified specific evidence gaps, particularly with regard to the value of follow up beyond three months for the longer term maintenance of physical activity. The lack of evidence relating to follow-up is particularly important as the literature suggests that approximately half of those who initiate a physical activity program relapse and return to their previous sedentary lifestyle within six months [14]. Yet maintenance of increased physical activity levels is essential to achieve the reported health benefits.

The Sheffield Physical Activity Booster Trial assesses the effectiveness and cost-effectiveness of Booster interventions to help people who have recently become more active maintain their increased physical activity levels. It is a three-arm, open-label, parallel group, randomised controlled trial (RCT) with a feasibility study. It compares two different intensities of a Motivational Interviewing (MI)[15] 'booster' intervention against no further intervention in middle-aged adults, aged 40 to 64, who have already increased their physical activity levels following a brief intervention. MI is one of the behaviour change interventions recommended by NICE for health promotion [16]. The purpose of the booster sessions is to help participants to sustain their physical activity levels and prevent relapse. The brief intervention involves provision of an interactive DVD based on a MI approach that is directive, person-centred and replicates the style of other successful behaviour change programmes [17] and was underpinned by all four constructs of the Trans-Theoretical Model of behaviour change [18]. All interventions are delivered by trained facilitators whose competence was independently assessed to ensure consistent delivery using a treatment fidelity framework [19,20].

The study is being carried out in two phases. An internal pilot and feasibility study has been undertaken in the first year to allow refinements to the sample size as well as to assess the feasibility of trial recruitment plans and the proposed interventions. This will be followed by the main study, which will evaluate the effectiveness and cost-effectiveness of "'mini" and "full" booster sessions, as an adjunct to a brief intervention, in sustaining physical activity in middle-aged adults. This paper only describes the results of the internal pilot and feasibility study.

## Methods

Full details of the research protocol have been published in another paper [21].

### Participants and setting

For the feasibility study, the local health services (NHS Sheffield) sent 3,300 letters with pre-paid reply cards to residents aged 40-64 yrs in one of the Enhanced Public Health Program (EPHP) areas of the city inviting them to enrol in a programme to help them get more physically active. This programme involved a "brief intervention" (interactive DVD), which is consistent with NICE guidance on physical activity and behaviour change interventions [13,16]. Although there are approx. 53,000 residents aged 40-64 yrs in the EPHP areas, this initial recruitment mail-out, represents approximately 10% of the 30,000 expected to be contacted for the main trial. All respondents' current physical activity levels were assessed; those not achieving the current recommended activity level (at least 30 minutes of moderate activity on at least 5 days [11]) and wishing to have support to become more active were eligible for the brief intervention. As the eligible respondents were sedentary at first contact, any increase in physical activity, irrespective of intensity, is likely to be beneficial. Thus, the DVD focussed on increasing activity in general, rather than emphasising moderate or strenuous activity specifically. Three months after initially receiving the brief intervention, DVD recipients' physical activity levels were re-assessed; those who had increased their physical activity level by at least 30 minutes of moderate or vigorous activity per week and were capable of giving written informed consent were invited to participate in the feasibility trial. Due to the practicalities of screening large numbers of respondents for both the brief intervention and subsequent trial, a self-report measure of physical activity, the Scottish Physical Activity Questionnaire (SPAQ)[22], was used at both of these stages.

### Interventions

Participants were randomly allocated to one of three groups:

1. "Full booster": two 20-30 minute face-to-face physical activity consultations using MI in a community setting, at one month and two months after randomisation, which aimed to promote and sustain change in physical activity status. This included an exploration of barriers and motives to change, decisional balance, agenda setting, action planning and relapse prevention;

2. "Mini booster": two 20-minute telephone based physical activity consultations (using MI) at one month and two months after randomisation. The mini-booster sessions also aimed to promote and sustain increased physical activity levels and focussed on exploration of relevant PA experience and commensurate action planning; or,

3. a control group, who received no additional support between randomisation and three month assessment.

The treatment fidelity and acceptability of the intervention will be reported elsewhere. In this report, the feasibility of the intervention is only considered in terms of intervention adherence (see below).

### Objectives

The primary objective of the main study is to determine whether physical activity, measured by accelerometry three months after randomisation (six months after a brief intervention), is significantly increased in participants allocated to two intervention groups ("full" or "mini" booster) compared to participants allocated to a control group. Both this and the other objectives of the main study are described in detail elsewhere [21].

The primary objective of the pilot study, however, is to assess the feasibility of both trial recruitment plans and the proposed interventions. Secondary objectives are:

1. To estimate the mailout response rate which is the proportion of individuals who responded, were contactable and were eligible for the brief intervention;

2. To estimate the effectiveness of the brief intervention, which is the proportion of recipients who increased their physical activity after 3 months and were eligible for the RCT;

3. To estimate the consent rate which is the proportion of eligible recipients who consented to participate and be randomised to any of the three arms;

4. To estimate the proportion of participants randomised to booster interventions who received the interventions per protocol, defined as receiving both of the intended face to face or telephone sessions.

5. To estimate the proportion of participants who provided primary outcome data 3 months post randomisation.

6. To verify the accuracy and completeness of the data. This routine data management activity was not a protocol-specified outcome, but is reported here because the funding body and trial steering committee requested a full report due to concerns about the usability of one of the patient reported outcome measures, the Exercise Evaluation Randomised Trial (EXERT) questionnaire [23].

### Outcomes

The primary outcome for the main study is objective measurement of physical activity using Actiheart (CamNtech, Cambridge, UK), which provides both accelerometery-derived physical activity counts (PAC) per week and mean total energy expenditure (TEE) in kilocalories (kcal) per day. Secondary outcome measures for the main study are reported in detail elsewhere [21] but include self-reported physical activity using the SPAQ [22] and Exercise Evaluation Randomised Trial (EXERT) questionnaire [23].

The prospectively defined success criteria for the feasibility study were:

1. To recruit and randomise at least 60 participants to the pilot trial (10% total sample size required for main study) and for at least 45 of these (75% retention) to provide 3 month follow-up measurements including accelerometry on the basis of an initial mail-shot to 3,000 individuals;

2. At least 70% of those randomised to the booster interventions receive the interventions per protocol, confirming the feasibility of the intervention;

3. On the basis of the pilot primary outcome (accelerometry) data collected, the sample size for the main trial will be re-calculated. The trial will not proceed if the revised sample size calculation suggests a total sample size notably greater than 600 will be required. Assuming the protocol and intervention remain unchanged, the participants recruited during the feasibility phase will be included in the full trial population.

Other objectives of the pilot trial were to obtain an estimated standard deviation for the primary outcome from the feasibility study data, to perform a power calculation for the main study for both mean TEE (kcal) per day and PAC per week, assuming a fixed sample size of 600 for the main comparison (combined interventions versus control), and to verify the accuracy and completeness of the data collected, assessed through 10% source data verification and prospectively defined as an acceptable error rate of 0.5%.

### Proposed sample size for the main study

The primary outcome for the main study is seven-day accelerometric assessment of physical activity (PAC per week) at three months post-randomisation. The original sample size calculation assumed that a mean difference of 400,000 PAC per week between the intervention and control groups at three months was the smallest clinically and practically important difference and that the SD of this outcome was 1.2 million counts per week. Hence with 450 participants (300 intervention: 150 control), the main trial was determined to have 90% power to detect this mean difference or greater between the "booster" and control arms as statistically significant at the 5% (two-sided) level using a two independent samples *t*-test. With 300 participants in the booster intervention (150 mini: 150 full booster) the trial would also have approximately 80% power to detect a similar mean difference of 400,000 counts per week between the two booster arms as statistically significant at the 5% (two-sided) level using a two independent samples *t*-test. Assuming an approximate 25% loss to follow-up by three-months, we proposed to recruit and randomise 200 participants per intervention group to give a total sample size of 600 participants.

### Statistical methods

The intention-to-treat (ITT) dataset included all participants who: (1) were randomised according to the allocation schedule (ignoring non-compliance, protocol deviations and withdrawals); and, (2) had a valid three-month accelerometry data defined as at least four complete days (out of seven) data. To minimise missing data, the "auto-fill" option on the Actiheart software, which fills gaps of up to two hours with the average values calculated from the recorded portion of the same day, was used. Days with more than two continuous hours of missing data, were classified as incomplete. Where missing data was determined to be because the participant had taken the accelerometer off to sleep, "sleeping" values were imputed. The results are reported based on standardised daily measurements (≥4 complete days).

The mean difference and standard deviations were used to estimate a minimum clinically important difference (MCID) based on 1/3 of the SD of physical activity counts per week. The sample size for the main study was recalculated, using the standard deviation of PAC per week from the pilot study and the original standardised effect size of 0.33 from initial sample size calculation. A power calculation for the main study was performed on both the original (PAC per week) and proposed (TEE per day) primary endpoints, based on their respective MCID and observed standard deviation from the feasibility study. This was only done for the main comparison (combined interventions versus control). When re-estimating the sample size using data from an internal pilot study the revised sample size estimate either stays the same or increases, it cannot be less than the original estimate [24,25].

### Ethical aspects

North Sheffield Research Ethics Committee granted ethical approval on 11^th ^February 2009 and informed consent was conducted as per the main study.

## Results

### Recruitment

**Figure 1:**
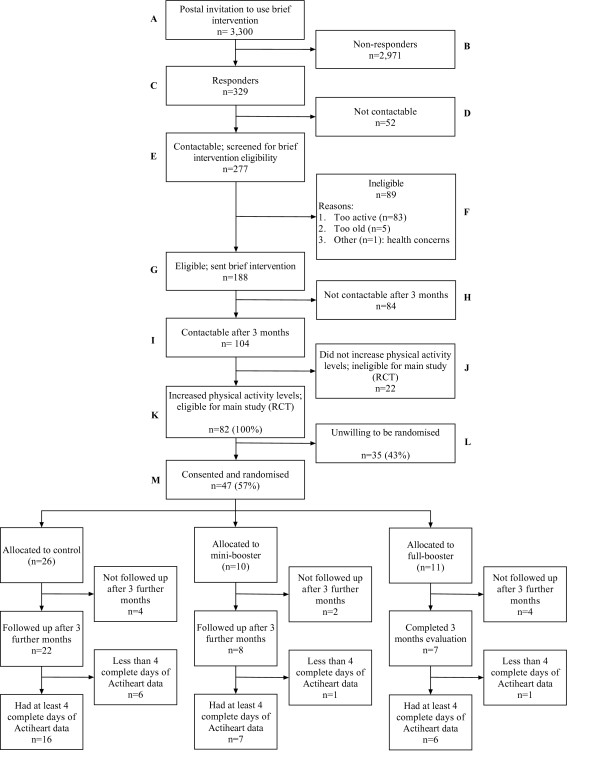
**Participant recruitment flowchart**.

Figure 1 shows the flow of participants through the feasibility study. Of the 3,300 people invited to apply for the interactive DVD in May/June 2009, 329 responded and 277 of these were contacted successfully. Of those contacted, 188 were achieving less than 30 minutes of moderate activity on at least 5 days a week, and so were eligible for the brief intervention (interactive DVD) by September 2009. Of those sent the DVD, 104/188 were still contactable after 3 months and of these, 82/188 (44%) had successfully increased their physical activity, making them eligible for the feasibility study. Forty-seven of the 82 (57%) eligible participants were recruited, consented and randomised to the feasibility study in November/December 2009, against a target of 60 participants, which translates as a ratio of 0.78 for the planned to actual recruitment targets. The main reason for non-participation given by eligible participants who declined to be randomised was that they were not interested in receiving further support to stay active (n = 18). Other reasons included lack of time (n = 6), family reasons (n = 3) and health concerns (n = 1). The remaining seven gave no specific reason for choosing not to be randomised.

### Baseline population characteristics

Table 1 shows the demographic characteristics of randomised participants. The figures for the baseline increase in physical activity show that some participants decreased their physical activity levels, suggesting that they may not have been eligible for the trial. Indeed two participants did not show a 30 minute increase in activity on the SPAQ at pre-trial screening. Both of these individuals, when they completed their initial SPAQ, at brief intervention screening, indicated that they had done more physical activity in the last week than normal. After accounting for the additional activity, both candidates were deemed to have increased their average physical activity levels by 30 minutes or more and were considered eligible.

**Table 1:** Baseline characteristics of randomised participants

Variable		n	mean	sd	minimum	maximum
Age (yrs)		47	56.2	6.7	41.1	65.2
SPAQ Change*		47	245.4	247.9	-30.0	1300.0
Height (m)		47	1.7	0.1	1.5	1.9
Weight (kg)		47	83.8	19.1	52.0	160.0
BMI (kg/m^2^)		47	30.4	5.6	20.3	46.0

		**n**	**%**			

Sex	*Male*	15	32			
	*Female*	32	68			
		47	100			
						
Martial	*Single*	2	4			
Status	*Married*	36	77			
	*Co-habiting*	3	6			
	*Divorced/Separated*	4	9			
	*Widowed*	2	4			
		47	100			
						
Ethnicity	*White-British*	46	98			
		1	2			
		47	100			
						
Employment	*Part-time*	12	26			
Status	*Full-time*	14	30			
	*Retired*	13	28			
	*Looking after family/home*	2	4			
	*Permanently sick/disabled*	2	4			
	*Temporarily sick/disabled*	1	4			
	*Unemployed actively seeking work*	1	2			
	*None of the above*	1	2			
		47	100			

* Difference between SPAQ at first contact and three months later (one week before randomisation). Two patients recorded a decrease in physical activity over 3 months, as measured on the SPAQ, of 25 and 30 minutes respectively. See text for explanation as to their inclusion.

### Validity of data and accuracy of the data entry

Acceptable error rates for data entry were prospectively defined as 0.5% in line with industry standards for phase I-III clinical trials. Error rates for two patient-reported outcome measures were initially considered unacceptable: the SPAQ (error rate 6.5%) and EXERT (error rate 1.3%). Providing the research assistants with additional training regarding data entry procedures eliminated the error rates for the SPAQ, but not for the EXERT, where error rates remained unacceptably high (2.1%). The errors are largely random and unlikely to be resolved by further training.

### Evaluable data at 3 months of follow-up

Thirty-seven (79%) of the 47 randomised participants had follow-up data at 3 months. Of the remaining ten, eight withdrew from the study, one died and one was lost to follow-up. Thirty-four (92%) of the 37 participants who provided 3 month data had at least one complete day of accelerometry data (two missing due to monitor set-up error and one removed due to skin reaction). Twenty-nine participants (62% of those randomised) had at least 4 complete days of accelerometry data (see Figure 1).

### Interventions received per protocol

Of the 21 participants who were randomised to the two booster interventions, 18 (86%) received their interventions as per protocol (10 and 8 in mini and full booster respectively) against a target of at least 70%.

### Summary of outcome measures using the Actiheart

The original sample size calculation of 450 participants with valid outcome data at 3 months post-randomisation, was based on detecting a standardised effect size of 0.33 or a mean difference of one-third of a standard deviation in the outcome measures between the booster and control groups. This equates to an estimated mean difference between the booster and control groups, based on the the observed standard deviation from the feasibility study, of 34,465 PAC per week and 102 kcal per day for TEE. For the TEE outcome a difference of 102 kcal is approximately 5% of mean TEE per day. Table 2 gives a summary of the Actiheart outcome measures, PAC and TEE, for the 29 patients with at least 4 complete days of accelerometry measurements. The mean PAC per week was 285,918 (SD 103,394) and the mean TEE per day was 2125 kcal (SD 305 kcal).

**Table 2:** Summary of primary outcome measures at 3-months post-randomisation

Outcome	N ^a^	Min	max	mean	Sd	p25	Median	p75	Effect size ^b^
PAC per week	29	109,008.0	633,024.0	285,917.9	103,394.0	228,528.0	269,568.0	329,184.0	34,464.7
									
Mean TEE (KCal) per day	29	1572.0	2761.7	2124.9	304.6	1917.7	2091.6	2300.0	101.5

^a ^only participants with at least 4 complete days (8 excluded; 5 had less than 4 complete days and remaining 3 had no Actiheart data at 3 months).

^b ^Effect size based on (1/3) of the estimated standard deviation (sd) from the data.

### Sample size re-calculation

The original sample size calculation assumed that physical activity would be measured using a simple hip-mounted accelerometer. It was also assumed that a mean difference of 400,000 PAC per week between the intervention and control groups at three months was the smallest clinically and practically important difference and that the SD of this outcome was 1.2 million counts/per week. Hence with 450 participants (300 intervention: 150 control), the main trial was determined to have 90% power to detect this mean difference or greater between the intervention and control arms as statistically significant at the 5% (two-sided) level using a two independent samples *t*-test. Assuming an approximate 25% loss to follow-up by three-months, it was proposed to recruit and randomise 200 participants per group giving total sample size of 600. Table 2 shows that the Actiheart accelerometer measures physical activity counts per week on a different scale of magnitude to a simple hip mounted accelerometer with a considerably lower mean and standard deviation.

When re-estimating the sample size using data from an internal pilot study the revised sample size estimate either stays the same or increases (it cannot be less than the original estimate) [25,26]. The original sample size calculation of 450 participants with valid outcome data at 3 months post-randomisation, was based on detecting a standardised effect size of 0.33 or a mean difference of one-third of a standard deviation in the outcome measures between the intervention and control groups. This equates to an estimated mean difference between the Booster and Control groups, based on the observed standard deviation from the feasibility stage of 34,465 PAC per week and 102 kcal per day for TEE.

To have a 90% power of detecting a mean difference of 102 kcal in mean TEE per day is between the groups would require 426 participants in total with evaluable data (control = 142; intervention = 284). Similarly, the total required sample size under the above conditions to detect a mean difference of a 34,465 PAC per week is 429. Therefore since the re-estimated sample size, of around 430 participants, is lower than the original estimate of 450 participants the trial will proceed with the original sample size estimate of 450 participants with evaluable data.

### Power estimation

The original sample size calculations assumed an approximate 25% loss to follow-up by three-months, and proposed to recruit and randomise 200 participants per intervention group giving a total sample size of 600 participants, to give 450 participants with evaluable data. The feasibility study, however, observed an attrition rate of approximately 38% with only 62% (29/47) randomised participants having complete accelerometry data. If this attrition rate is repeated in the main study then with an initial sample size of 600 and an attrition rate of 40% and hence 360 participants with valid 3-month post randomisation accelerometry data then the study would have 85% power to detect a mean difference of 102 kcal in TEE between the groups, if such a difference truly exists. If this 40% attrition rate is observed in the main study then we believe that the marginal loss in power is acceptable and propose to carry on recruiting and randomise 200 participants per intervention group to give a total sample size of 600 participants.

### Serious adverse events

There were two serious adverse events, neither of which were considered to be related to the intervention. In the first, a participant died of Myocardial Infarction and Ischemic Heart Disease prior to receiving the intervention. In the second, a participant was admitted to hospital with a suspected Myocardial Infarction. Symptoms came on at rest. Tests revealed that it was not a Myocardial Infarction, and was most likely to be angina-related pain.

## Discussion

### Summary of findings

Forty-seven participants were recruited (78% target), thirty-seven were retained at three months and 29 of these provided at least four days accelerometry data (64% target). The number receiving the Booster interventions per protocol surpassed the feasibility target and data error rates were within acceptable limits, with the exception of the EXERT questionnaire.

From the data recorded, the standard deviation is estimated to be 103,394 PAC per week or 305 kcal per day for TEE and the sample size has been recalculated. With 600 randomised participants and 30% loss to follow-up the study will have approximately 90% power to detect a mean difference of 102 kcal per day in TEE between the groups as statistically significant at the 5% two-sided level. The observed attrition rate in the pilot study was, however, higher than this at 38%. With an initial sample size of 600 and assuming an attrition rate of 40%, and hence 360 participants with valid accelerometry data at three months then the study would have 85% power to detect a mean difference of 102 kcal in TEE between the groups.

### Improving recruitment and retention

The fact that 82 out of the 104 DVD recipients who were screened after three months had increased their physical activity and were eligible for the study is promising. But, as only 47 were consented and randomised, strategies to improve recruitment are clearly required. The initial invitation to use the brief intervention (DVD) is being extended to all 40-64 year olds resident in EPHP areas giving a pool of 53,000 rather than only 30,000 residents (as originally intended) from which to draw (Figure 1 Box A). Extrapolating from the pilot data and with other things being equal, we would now expect the proposed mailout to 30,000 residents to result in 420 (95% CI: 380 to 570) participants randomised to the main trial (Figure 1 Box M), somewhat short of the proposed 600. With a mailout of 50,000, however, we would expect to be able to recruit 700 (95% CI: 550 to 950) participants. We have also increased the intensity with which the study is promoted. In the pilot area, the study was implemented without NHS or community-based support, awareness raising or publicity. Knowledge of the brief intervention's availability was communicated purely by one-to-one postal contact. In future areas, the invitation letters are being supported by local awareness raising involving EPHP area leads and other NHS links, community activists and wider media promotion.

A large minority (30%) of those who responded to the initial invitation and were contactable (Figure 1 Box F) were ineligible for the brief intervention almost exclusively because they were already too active. While it is too late to alter the recruitment materials for this study, any future trial attempting to identify people with sedentary lifestyles might consider incorporating a single item measure of current physical activity into the reply card, to avoid unnecessary telephone screening.

Further down the CONSORT diagram, many of those who were sent the brief intervention were not contactable for pre-trial screening after three months (Figure 1 Box H). To address this, during initial screening preferred method of contact is now confirmed and SMS messaging or e-mail offered, as well as post and telephone.

The refusal of consent by 43% of those eligible to be randomised is unsurprising considering the recruitment yield of prevention trials generally, e.g. Spilker and Kramer cite typical recruitment yields of only 1% to 6% [27]. Putting recruitment figures in context is not easy as many trial reports do not record how many people were invited to participate, only how many were screened and randomised. For instance, the EXERT trial, which evaluated GP referral for different exercise programmes, report randomising 949 out of 1105 people who contacted the team, but did not record the number of patients who were advised by their GPs to take part in the programmes, but did not wish to do so [23]. Korde eventually randomised 29 out of 352 women (8.2%) at risk of breast cancer who initially expressed interest in the study; it is not known how many women the team initially contacted [28]. Similarly, the BRUM-CHF trial evaluating home-based exercise rehabilitation for patients with congestive heart failure screened 1639 patients, invited 642 (39.2%) to participate and randomised 169 (10.3%) [29]. The SMART trial, evaluating different modes of self-monitoring on short- and long-term weight loss, finally randomised 210/704 (29.8%) originally screened for eligibility by phone but again there is no information about the numbers contacted in the original mailout [30]. The numbers in these studies further suggests that converting eligible to randomised participants is unlikely to be easy. There is little difference between the recruitment rates of individual research assistants working on Booster, so training is unlikely to be an issue. Where reasons have been given for non-consent, the most frequently cited is lack of interest in further support to stay active.

Of the 47 participants randomised into the trial, ten did not complete three month follow up assessments. Eight of these participants had withdrawn prior to the three month assessment, with the most common reason cited as being too busy to continue (n = 5). Strategies to minimise numbers withdrawing are needed. During the feasibility phase, both intervention and assessment sessions were being delivered at venues local to the participant's residence with appointments scheduled at the participant's convenience, including in the evening and at weekends. During the main trial, however, sessions will also be offered at venues which are close to large employment hubs. It is hoped that this wider choice of venue will make the sessions more readily accessible to participants. Although it is not possible to abbreviate the intervention, it may be possible to shorten the assessment visits for those participants who express concerns about time constraints, e.g. by mailing questionnaires out in advance, to be returned at a shortened assessment visit.

Three Actiheart devices were returned with no usable data Two of these cases were due to set-up error resulting in the device not recording. This is a training issue and all research assistants will be given refresher training on programming the devices prior to the main trial to minimise the risk of similar set-up errors occurring in future. The remaining device with no data was due to removal soon after fitting due to skin irritation. This is an acknowledged issue in research using Actiheart devices: during fitting the participant's skin is cleaned and abraded to remove the top layer of skin prior to the application of the two self-adhesive ECG pads which the Actiheart device attaches to. The process of cleaning and abrasion combined with the presence of the ECG pads can cause skin irritation in some participants. Previous studies using Actiheart devices have reported skin irritation rates in up to 12% participants for protocols requiring seven days continuous wear [31]. Five devices had less than four full days data; there was no specific reasons were identifiable for this. As some initial data was recorded in all cases and the Actihearts all recorded for the full seven days, it is likely that participants removed the monitors before the end of the monitoring period but did not report that fact. Examination of the assessment records did not reveal any systematic failure to provide full data sets, e.g. due to a particular research assistant delivering instructions. During the main trial, however, additional emphasis will be placed on ensuring that participants understand the importance of wearing the monitor for the full monitoring period. Spare ECG pads will also be provided in case shorter periods of wear were due to the original set of pads becoming detached from the skin.

### Reach and representativeness

Glasgow and colleagues define 'reach' as an individual-level measure of participation, which refers to the percentage and risk characteristics of persons who receive or are affected by a policy or program [32]. A casual glance at the CONSORT flow diagram (Figure 1), may lead to the conclusion that this intervention is lacking the reach necessary for an effective health behaviour intervention. Study eligibility criteria, however, means that the recruitment yield should be understood as 57% of those having received and benefited from a brief intervention (Figure 1 Box K) rather than 25% of those eligible for the brief intervention (Box G), or .4% of those originally contacted (Box A).

The other concern is whether the study population is representative of target populations. The high number of women (68.1%) in the trial population reflects those who were eligible for the brief intervention (Figure 1 Box G, 68.5% female) and those who came forward for the brief intervention (Figure 1 Box E: 63.5% female) rather than the target population of the pilot area, High Green (50.5% female). The age distribution of the pre-trial population (Figure 1 Box E: n = 277) was not only skewed but differed markedly between genders. Women showed a linear increase in the response to the initial mailout with increasing age, such that almost twice as many women in the 60-64 years bracket responded as those aged 40-44 years. On the other hand, the response to the mailout among men was bimodal with peaks in the 40-44 years and 60-64 years categories and the fewest respondents from the 50-54 years category. High Green residents are predominantly White-British (97.4%) and this seemed to be reflected in both our contactable respondents and those eligible for the DVD, although no ethnicity data was collected during screening as it was not relevant to eligibility. Similarly, no income data was collected on the pre-trial population. Concerns expressed by local public health leads, however, that a mass mailout would attract people from the relatively wealthy periphery rather than those in greater need living in the more deprived core of the neighbourhood appear unwarranted; an informal mapping exercise revealed a relatively even spread over the pilot area.

### Proposed change of primary outcome

As mentioned above, a change in how the primary outcome, physical activity, is quantified in this trial has been proposed and was presented to representatives of the funding body prior to three month data collection or analysis for the pilot study. The proposal was put to independent scientific review and conditionally agreed before the statistical analysis plan for the feasibility analysis was signed off and analysis commenced. Further details of the rationale behind this change are available from the lead author and a brief summary has been published elsewhere [21].

### Data quality and the EXERT questionnaire

There were problems with the questionnaire adopted from the EXERT trial [23] with persistent, mostly random, errors occurring in the data despite retraining of research assistants. The EXERT trialists had adapted the this questionnaire from another physical activity trial in West London [33], which itself had been adapted from a self-administered seven day recall questionnaire used in other community intervention studies [34]. Although used to measure changes in physical activity over time in EXERT, the original purpose of the West London questionnaire was to classify individuals as active or inactive vis-à-vis existing recommendations on physical activity and health [33]. Discussions with the EXERT trial team established that a simpler questionnaire would have been preferable for both trial participants and the analysis team. Furthermore, some elements did not look reliable in the EXERT trial and not all of the data collected was used in the final analysis (Julia Critchley and Tony Isaacs, personal communications). Thus, the Booster trial steering committee have advised abandoning the EXERT questionnaire.

## Conclusions

Although the response to the brief intervention was promising, there were problems converting eligible individuals to randomised participants. Retention of sufficient participants with evaluable data was also an issue. Changes in recruitment and retention strategies are needed to ensure adequate numbers are randomised and retained. There are also some issues surrounding data collection to address. The sample size re-calculation confirmed the original sample size of 600 to be appropriate and that, with 600 participants, the trial will still have sufficient power to detect a significant difference between groups despite the greater than anticipated attrition rate.

## Competing interests

The authors declare that they have no competing interests.

## Authors' contributions

EG, RJC, JDB, HC, SJW and CLC conceived, designed and secured funding for the trial; EJS and DH contributed to further protocol development and coordinated the trial; AL provided data management for the trial; MD wrote the statistical analysis plan, advised by SJW, and conducted the statistical analysis; JDB trained and supervised the RAs in delivering the MI intervention. All authors read and approved the final manuscript.

## Pre-publication history

The pre-publication history for this paper can be accessed here:

http://www.biomedcentral.com/1471-2458/11/129/prepub
